# Detection of Synchronous Parathyroid Adenoma and Breast Cancer with ^18^F-Fluorocholine PET-CT

**DOI:** 10.1007/s13139-015-0357-x

**Published:** 2015-08-21

**Authors:** Wessel MCM Vorselaars, Wouter P. Kluijfhout, Menno R. Vriens, Carmen C. van der Pol, Inne HM Borel Rinkes, Gerlof D. Valk, Bart de Keizer

**Affiliations:** Department of Oncological and Endocrine Surgery, University Medical Center Utrecht, Utrecht, The Netherlands; Department of Endocrine Oncology, University Medical Center Utrecht, Utrecht, The Netherlands; Department of Nuclear Medicine and Radiology, University Medical Center Utrecht, Heidelberglaan 100, 3584 CX Utrecht, The Netherlands

**Fig. 1:**
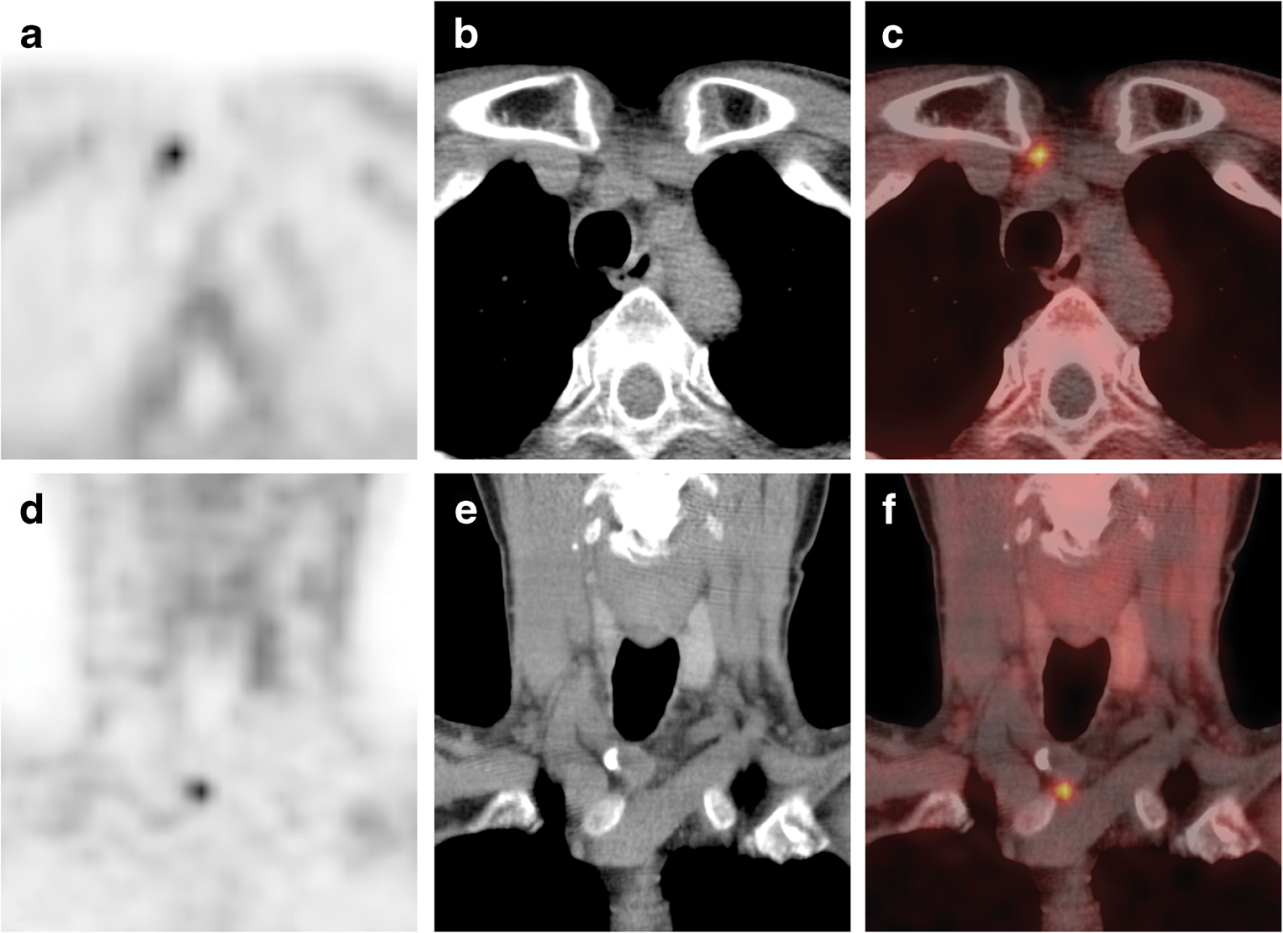
^18^F-Fluorocholine PET-CT of the neck/mediastinum: axial (**a**-**c**) and coronal (**d**-**f**) PET, CT and fused PET-CT images showing a focal uptake (SUVmax = 1.8) at the lower anterior neck, level VI, anterior to the right common carotid artery, suggesting parathyroid adenoma

**Fig. 2:**
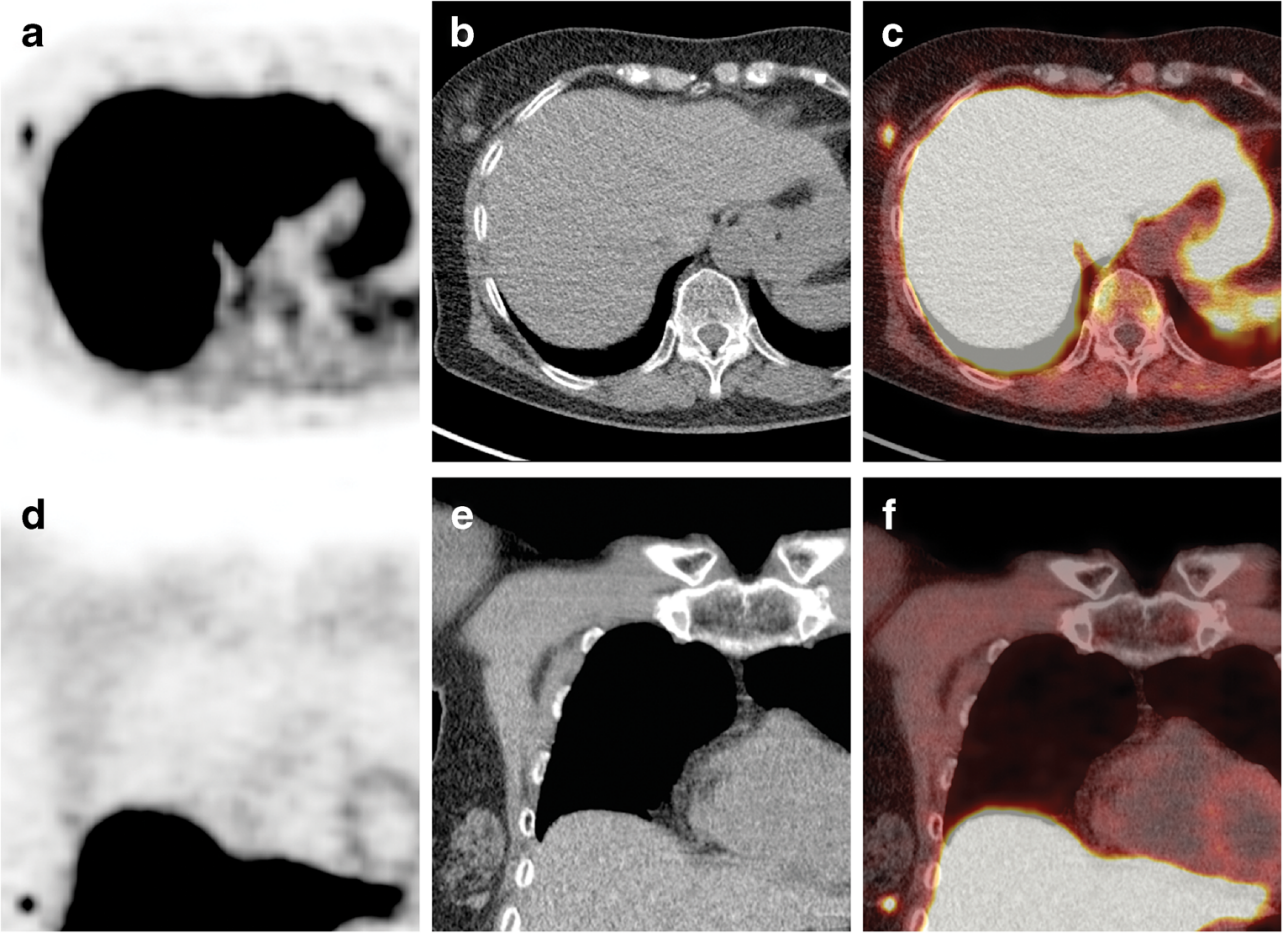
^18^F-Fluorocholine PET-CT of the neck/mediastinum: axial (**a**-**c**) and coronal (**d**-**f**) PET, CT and fused PET-CT images showing an additional focal uptake (SUVmax = 2.5) in a nodal structure of 1.2 cm located in the lateral lower quadrant of the right breast

A 71-year-old woman was referred to our tertiary care center for evaluation of asymptomatic recurrence of primary hyperparathyroidism. As per our protocol, the patient underwent neck/mediastinum ^18^F-fluorocholine (FCH) positron emission tomography-computed tomography (PET-CT) for localization. In our institution, FCH PET-CT is performed in patients with hyperparathyroidism and negative conventional imaging [1]. FCH PET-CT is a promising new imaging modality for detection of hyperfunctioning parathyroid glands [2, 3]. Thirty minutes after injection of 139 MBq (3.8 mCi), the PET-CT images showed a focal uptake (SUVmax = 1.8) at the lower anterior neck, level VI, anterior to the right common carotid artery (Fig. 1a-f), suspicious for parathyroid adenoma. Additionally, it showed a second focal uptake (SUVmax = 2.5) in a nodal structure measuring 1.2 cm, within the outer lower quadrant of the right breast (Fig. 2a-f). No other pathological uptake was seen. On the subsequent ultrasound (US) of the breast, there was an area of microcalcifications seen with no definite abnormal lesion. US of the axilla was negative for any suspicious lesions. Mammography showed a blurry 1.3-cm mass without clear boarders and microcalcifications within relatively dense fibro-glandular tissue (BI-RADS IV). US-guided core biopsy showed papillary carcinoma. Pathological examination after breast-conserving surgery revealed papillary breast cancer of 1.0 cm, staged pT1N0. The location of the cancer corresponded with that indicated by FCH PET-CT. Due to the finding of breast cancer, surgery for primary hyperparathyroidism was postponed.

Currently, mammography and US are considered the standard of care in the preoperative workup of breast cancer [4]. Multiple other imaging modalities, including positron emission mammography (PEM) with a variety of radionuclide tracers as well as magnetic resonance imaging (MRI), are currently under investigation [5–7]. Breast cancer has previously been found to have increased uptake of choline [8]. Therefore, the use of ^11^C-choline PET/CT has been used to accurately localize these malignant tumors [9]. One major drawback is that the half-life of ^11^C-methionine is only 20 min. This requires on-site production of the tracer to be used in the study, thereby strongly limiting its clinical applicability. The half-life of FCH is 110 min, enabling off-site production and distribution, making it much more practical to use as an imaging modality in this context [10]. Furthermore, FCH is already being used in the evaluation of prostate cancer and is therefore more widely available than other radiotracers [11, 12].

As can be seen in the case presented, high FCH uptake was seen in a small breast cancer. Due to its favorable half-life and wide availability by its use as a localization technique for patients with prostate cancer and complicated hyperparathyroidism, FCH PET-CT may be a new promising modality in the imaging of breast cancer.
